# Role of psychosocial factors on the effect of physical activity on physical function in patients after lumbar spine surgery

**DOI:** 10.1186/s12891-021-04622-w

**Published:** 2021-10-18

**Authors:** Hiral Master, Renan Castillo, Stephen T. Wegener, Jacquelyn S. Pennings, Rogelio A. Coronado, Christine M. Haug, Richard L. Skolasky, Lee H. Riley, Brian J. Neuman, Joseph S. Cheng, Oran S. Aaronson, Clinton J. Devin, Kristin R. Archer

**Affiliations:** 1 Department of Orthopaedic Surgery, Center for Musculoskeletal Research, Vanderbilt University Medical Center, 1215 21st Ave South, Nashville, TN 37232 USA; 2 Vanderbilt Institute for Clinical and Translational Research, Vanderbilt University Medical Center, Nashville, TN USA; 3 Department of Health Policy & Management, Johns Hopkins Bloomberg School of Public Health, Baltimore, MD USA; 4 Department of Physical Medicine and Rehabilitation, Johns Hopkins Medicine, Baltimore, MD USA; 5 Department of Physical Medicine and Rehabilitation, Osher Center for Integrative Medicine, Vanderbilt University Medical Center, Nashville, TN USA; 6 Department of Orthopaedic Surgery, Johns Hopkins Medicine, Baltimore, MD USA; 7 Department of Neurological Surgery, University of Cincinnati College of Medicine, Cincinnati, OH USA; 8 Howell Allen Clinic, Saint Thomas Medical Partners, Nashville, TN USA; 9 Steamboat Orthopedic and Spine Institute, Steamboat Springs, CO USA

**Keywords:** Walking, Steps per day, Function, Depression, Self-efficacy, Fear, Spine surgery, Degenerative lumbar spine conditions, Patient-reported outcomes

## Abstract

**Background:**

The purpose of this study was to investigate the longitudinal postoperative relationship between physical activity, psychosocial factors, and physical function in patients undergoing lumbar spine surgery.

**Methods:**

We enrolled 248 participants undergoing surgery for a degenerative lumbar spine condition. Physical activity was measured using a triaxial accelerometer (Actigraph GT3X) at 6-weeks (6wk), 6-months (6M), 12-months (12M) and 24-months (24M) following spine surgery. Physical function (computerized adaptive test domain version of Patient-Reported Outcomes Measurement Information System) and psychosocial factors (pain self-efficacy, depression and fear of movement) were assessed at preoperative visit and 6wk, 6M, 12M and 24M after surgery. Structural equation modeling (SEM) techniques were utilized to analyze data, and results are represented as standardized regression weights (SRW). Overall SRW were computed across five imputed datasets to account for missing data. The mediation effect of each psychosocial factor on the effect of physical activity on physical function were computed [(SRW for effect of activity on psychosocial factor X SRW for effect of psychosocial factor on function) ÷ SRW for effect of activity on function]. Each SEM model was tested for model fit by assessing established fit indexes.

**Results:**

The overall effect of steps per day on physical function (SRW ranged from 0.08 to 0.19, *p*<0.05) was stronger compared to the overall effect of physical function on steps per day (SRW ranged from non-existent to 0.14, *p*<0.01 to 0.3). The effect of steps per day on physical function and function on steps per day remained consistent after accounting for psychosocial factors in each of the mediation models. Depression and fear of movement at 6M mediated 3.4% and 5.4% of the effect of steps per day at 6wk on physical function at 12M, respectively. Pain self-efficacy was not a statistically significant mediator.

**Conclusions:**

The findings of this study suggest that the relationship between physical activity and physical function is stronger than the relationship of function to activity. However, future research is needed to examine whether promoting physical activity during the early postoperative period may result in improvement of long-term physical function. Since depression and fear of movement had a very small mediating effect, additional work is needed to investigate other potential mediating factors such as pain catastrophizing, resilience and exercise self-efficacy.

**Supplementary Information:**

The online version contains supplementary material available at 10.1186/s12891-021-04622-w.

## Introduction

Over the past two decades, the rate of spine surgery for lumbar degenerative conditions has doubled in the United States [1, 2] and is associated with high hospital costs, i.e., over $10 billion in 2015 [3]. Despite surgical intervention, postoperative physical activity remains stagnant or decreases [4–7]. Physical activity has the potential to be an important target for rehabilitation efforts, with recent work demonstrating that engaging in more physical activity (i.e., walking) early after surgery has a positive impact on disability and opioid use up to 12 months after lumbar spine surgery [8, 9]. Further, engaging in physical activity is related to better physical functioning in older adults with and without pain [10–13]. At present, little is known about the longitudinal relationship between physical activity and physical function in patients after lumbar spine surgery. Some research suggests that limited physical functioning may impair an individual’s ability to participate in physical activity [14, 15]. A better understanding of the directional relationship between physical activity and physical function will provide insights on the early postoperative treatment strategies needed to maximize patients’ long-term recovery trajectory.

Psychosocial factors such as fear of movement and self-efficacy are important determinants of physical activity and physical function after lumbar spine surgery [16, 17]. Longitudinal studies have found postoperative depression and fear of movement to be significant contributors of disability and physical function outcomes [18–21], while low self-efficacy has demonstrated a negative effect on disability and physical activity [22, 23]. In other chronic pain and older adults populations, these psychosocial factors have been found to be important mediators of pain on function [24, 25] and physical activity on function [26]. Mullen et al. found that walking more increased walking self-efficacy, which in turn reduced the risk of lower extremity functional limitation in older adults [26]. Mediation analyses are warranted in the spine surgery population to help inform the prioritization of mental health assessment and management.

The purpose of this study was to investigate the longitudinal postoperative relationship between physical activity, psychosocial factors, and physical function in patients undergoing lumbar spine surgery. Based on prior work in older adults [26, 27], we hypothesized that the relationship of early postoperative physical activity to long-term postoperative function after lumbar spine surgery would be stronger compared to the relationship of function to activity. Further, we hypothesized that postoperative psychosocial factors of pain self-efficacy, depression, and fear of movement would mediate the relationship between physical activity and physical function.

## Methods

### Study participants

Two hundred and forty-eight English-speaking adults undergoing lumbar spine surgery were recruited from two academic medical centers in the United States. The detailed description of the recruitment of the study participants has previously been published [28]. Patients were included in the study if they underwent a laminectomy and/or arthrodesis procedure for a lumbar degenerative condition, including spondylosis, spondylolisthesis, and spinal stenosis. Participants were excluded if they were primarily undergoing discectomy or revision procedures, had a history of neurological or psychotic disorder, had a workmans’ compensation claim, or were not able to return to the clinic for a post-operative follow-up visit.

### Design and procedures

**Fig. 1:**
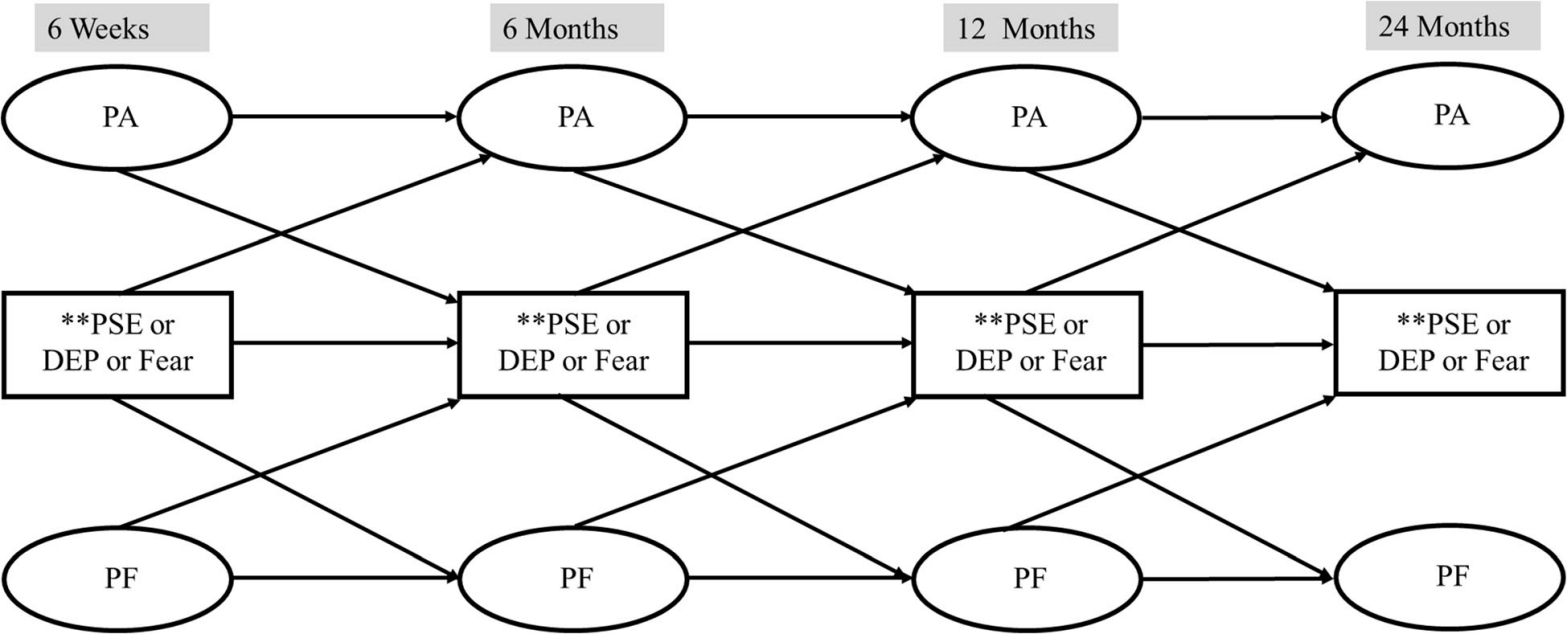
Structural equation model for postoperative recovery after lumbar spine surgery. PA = physical activity as measured by accelerometer (average steps per day); PF = Physical function as measured by PROMIS; PSE = Pain self-efficacy as measured by Pain Self-Efficacy Questionnaire; Dep = Depressive symptoms as measured by Patient Health Questionnaire-9; Fear = Fear of movement as measured by 13-item Tampa Scale for Kinesiophobia. *SEM models controlled for age, employment, comorbidities, prior spine surgery, group, and preoperative pain-self-efficacy, depression, fear of movement, physical function, and back and leg pain. **PSE, DEP and Fear were explored in separate SEM models

This study is a secondary analysis of prospectively collected data from a randomized controlled trial (NCT 02184143) [28]. The original trial compared two different rehabilitation programs provided between six weeks and three months after surgery (cognitive-behavioral based physical therapy (CBPT) or education) and no significant outcome differences were found between the two post-operative rehabilitation programs [29]. Thus, data from the two groups (CBPT and education) were pooled for the current study in order to test a conceptual model of postoperative recovery after lumbar spine surgery (i.e., physical activity to physical function vs. function to activity; Fig. 1).

Institutional Review Boards at the two participating sites approved the study and all participants provided written informed consent prior to data collection. Participants wore accelerometers and completed questionnaires on physical function and psychosocial factors at 6-weeks, 6-, 12- and 24-months following lumbar spine surgery. A preoperative questionnaire was also completed by patients to collect information on demographic and clinical characteristics as well as pain, physical function, and psychosocial factors. Surgical information was abstracted from the medical record.

### Outcome measures

Physical activity was objectively quantified as steps per day, which was measured using an accelerometer (Actigraph GT3X). The Actigraph GT3X is a triaxial accelerometer that validly quantifies walking in free-living conditions [30, 31]. We used a publicly available accelerometer algorithm provided by the National Cancer Institute to process Actigraph data [32] and eliminated non-wear time using a validated algorithm in adults with knee osteoarthritis [33]. A participant was considered to have a valid wear day if the monitor was worn for at least 10 hours [32]. The number of steps was summed across all the valid wear days, and an average steps per day was calculated for each participant.

Physical function was assessed using the computerized adaptive test (CAT) domain version of Patient-Reported Outcomes Measurement Information System (PROMIS) [34, 35]. PROMIS utilizes a T score metric where 50±10 represents the general population mean and standard deviation. Each item is rated on a 5-point Likert scale and the total score ranges from 0 to 100. The PROMIS physical function domain is a valid and reliable tool in the spine surgery population [36–38], and higher PROMIS T-scores represent higher levels of physical functioning [34].

### Psychosocial variables

Three psychosocial variables were constructed to examine mediation between physical activity and physical function. The 10-item Pain Self-Efficacy Questionnaire (PSEQ) was used to measure the strength and generality of a person’s belief in his/her ability to accomplish a range of activities despite pain [39]. Each item is rated on a 7-point Likert scale from ‘not at all confident’ to ‘completely confident.’ The total score can range from 0 to 60 with higher numbers indicating higher pain self-efficacy. A score <40 on PSEQ has been used to determine the presence of low pain self-efficacy in chronic pain populations [22, 40]. The PSEQ has been found to have excellent internal consistency, good test-retest reliability, and construct validity through correlations with depression, anxiety, coping strategies, and pain in patients with chronic pain [40]. Patient Health Questionnaire-9 (PHQ-9) was used to assess signs and symptoms characteristic of depression [41]. Each of the 9 items is rated on a 4-point Likert scale with scoring ranging from “not at all” to “nearly every day”. The total score can range from 0 to 27 with higher numbers indicating higher depressive symptoms. A score ≥10 on PHQ-9 has been used to determine the presence of moderate to severe depressive symptoms [41]. The PHQ-9 has excellent reliability and is a sensitive measure of major depression in primary care and following spine surgery [42, 43]. The 13-item Tampa Scale for Kinesiophobia (TSK-13) was used to measure fear of movement [44]. Each item was rated on a 4-point Likert scale with scoring alternatives ranging from ‘strongly disagree’ to ‘strongly agree.’ The total score can range from 13 to 52 with higher numbers indicating higher fear of movement [44]. A score ≥33 on TSK-13 has been found to identify moderate to severe fear of movement in adults with chronic musculoskeletal pain [45]. The TSK-13 has good internal consistency and test-retest reliability in surgical patients and patients with various musculoskeletal conditions [44, 46, 47].

### Statistical analysis

Demographic and clinical characteristics and outcome and psychosocial scores of all the participants included in the analytical sample were described using mean and standard deviation for continuous variables, and frequency for categorical variables. Percentages were computed to investigate the proportion of the sample who had high psychosocial risk as defined by the PSEQ (<40), PHQ-9 (≥10), and TSK-13 (≥33) thresholds. All the descriptive statistics were calculated using SPSS software (IBM Inc, Chicago, IL). Structural equation modeling (SEM) techniques were employed to examine the longitudinal relationship between outcome measures (physical activity and physical function) and psychosocial variables (pain self-efficacy, depression, and fear of movement) over time (Fig. 1). SEM allows modelling for correlated residuals, thereby reducing the measurement error and accurately describing longitudinal models with repeated measures [48]. Thus, in all the SEM models, residual error terms for the different time points of the same measure were allowed to covary.

Four separate SEM models examined the longitudinal postoperative relationship over time between (i) outcome measures (physical activity and physical function), (ii) outcome measures and psychosocial variable (pain self-efficacy), (iii) outcome measures and psychosocial variable (depression) and (iv) outcome measures and psychosocial variable (fear of movement). Age, employment, comorbidities, prior spine surgery, and preoperative self-efficacy, depression, fear of movement, physical function, and back and leg pain were included as covariates in all models based on prior literature [8, 22, 49–51]. Separate bivariate analyses were conducted (results not shown) to confirm the association of covariates with physical activity and physical function. Correlation matrices were used to examine potential collinearity. Each SEM model was tested for model fit by assessing the established fit indexes, including the Comparative Fit Index (CFI), the Tucker-Lewis Index (TLI), Standardized root mean squared residual (SRMR) and the Root Mean Square Error of Approximation (RMSEA) [52–54]. CFI and TLI values greater than 0.9, SRMR value less than 0.08, and RMSEA value at least <0.08 but ideally <0.05 indicates an acceptable fit [55]. Stability index was computed by testing eigenvalue stability conditions [56]. The SEM model was considered stable if the stability index lies within the unit circle [56].

Missing data were handled with multiple imputation using predictive mean matching and five imputed datasets [57]. Approximately, 50% to 55% data were missing at 24 months while less than 15% of data was missing at prior time-points (see footnote in Table 2). Analysis for each SEM model was conducted on all five imputed datasets (presented as imputed models 1 through 5 and abbreviated as M1to M5 in Tables 3 to 7) with 248 participants. Specifically, in order to build a SEM model that has optimal model fit, we eliminated any insignificant relationships between covariates and outcomes of interest (activity and function), any insignificant covariances, and any insignificant associations between physical activity, psychosocial factors and physical function. The relationships that were inconsistent and non-significant were removed from the final SEM model and were stated as non-existent in the text and not statistically significant (n.s.) in the tables. The directionality between covariates, psychosocial factors and outcomes measures in the final SEM model were consistent with preliminary bivariate analyses that were separately conducted.

Standardized regression weights (SRW), 95% confidence intervals (CI) and p-values were computed for each imputed dataset and pooled estimates were obtained from the five imputed datasets using Rubin’s approach [58]. The SEM models were examined using maximum likelihood estimation and all the fit indexes and SRWs were calculated in STATA 12 [59]. We employed point estimation methods as suggested by MacKinnon et al [60] to compute the magnitude of the mediation effect. More specifically, the 6-month mediation effect of each psychosocial variable on the outcome measures were calculated by dividing the effect of activity at 6 weeks on psychosocial at 6 months and effect of psychosocial at 6 months on function at 12 months by the effect of activity at 6 weeks on function at 12 months (Formula 1). The 12-month mediation effect used a similar formula; however, 6 month, 12 month and 24 month timepoints were used instead of 6 weeks, 6 months and 12 months, respectively. Also, sensitivity analyses were conducted with complete data, which excluded the 24 month time-point, to confirm findings. Estimates were similar and only the imputed results are reported. Finally, E-values, which provide a useful measure of sensitivity to potentially unobserved confounders were computed for mediation co-efficients [61].

Formula 1: Computation for 6 month mediation effect.

$$ \frac{\left[\left(6\mathrm{Wk}\ \mathrm{physical}\ \mathrm{activity}\to 6\mathrm{M}\ \mathrm{psychosocial}\right)\times \left(6\mathrm{M}\ \mathrm{psychosocial}\to 12\mathrm{M}\ \mathrm{physical}\ \mathrm{function}\right)\right]}{6\mathrm{Wk}\ \mathrm{physical}\ \mathrm{activity}\to 12\mathrm{M}\ \mathrm{physical}\ \mathrm{function}}\times 100 $$

## Results

### Participant characteristics

Data from 248 participants (mean ± standard deviation; age = 62.2 ± 11.9 years; body mass index= 32.4 ± 6.6 kilogram/meter^2^ and 126 (50.8%) females) are presented in Table 1. One hundred and thirty-one (52.8%) participants reported at least one comorbidity. The average preoperative back and leg pain scores were 6.7 ± 2.3 and 6.5 ± 2.4, respectively. Table 2 shows the summary of steps per day for physical activity, PROMIS physical function, and PSEQ, PHQ-9, and TSK-13 scores for psychosocial variables over the course of the study. Before surgery, 77%, 36%, and 58% of patients were identified as having low-self-efficacy, moderate to severe depressive symptoms, and high fear of movement, respectively. Improvement was noted at 6-weeks after surgery, with 44% reporting low self-efficacy, 20% depressive symptoms, and 27% high fear of movement. Psychosocial risk at 6 and 12 months ranged from 33% to 37% for PSEQ, 11% to 14% for PHQ-9, and 14% to 15% for TSK-13. At 24-months, 11%, 0%, and 9% of patients reported PSEQ score < 40, PHQ-9 score ≥10, and TSK-13 score ≥33.

**Table 1:** Descriptive statistics of participants who were enrolled in the study (*N*=248)

Characteristics	N (%) or Mean ± SD
Age in years, Mean ± SD	62.2 ± 11.9
Female sex, N (%)	126 (50.8)
White race, N (%)	215 (86.7)
More than high school education, N (%)	182 (73.4)
Married, N (%)	164 (66.1)
BMI in kg/m^2^, Mean ± SD	32.4 ± 6.6
Employed prior to surgery, N (%)	91 (36.7)
1 or more co-morbid conditions, N (%)	131 (52.8)
Fusion surgery, N (%)	164 (66.1)
Prior spine surgery, N (%)	74 (29.8)
Preoperative back pain, Mean ± SD	6.7 ± 2.3
Preoperative leg pain, Mean ± SD	6.5 ± 2.4

*BMI* body mass index

**Table 2:** Descriptive statistics of steps per day, physical function and psychosocial factors (pain self-efficacy, fear of movement and depression) over time

		Post-surgery (Mean ± SD)			
Factors	Preoperative (Mean ± SD)	6-weeks	6-months	12-months	24-months
Steps per day^a^	-	3655 ± 2246	4106 ± 2446	3983 ± 2137	4011 ± 2406
PROMIS physical function^b^	33.5 ± 5.6	37.6 ± 7.9	42.3 ± 8.0	42.5 ± 8.6	43.0 ± 9.5
PSEQ^c^	30.0 ± 13.4	40.5 ± 14.7	46.2 ± 13.4	44.6 ± 14.6	47.7 ± 12.7
PHQ-9^d^	8.3 ± 5.4	5.3 ± 5.1	4.0 ± 4.4	4.4 ± 4.9	2.3 ± 2.5
TSK-13^e^	34.0 ± 6.7	28.4 ± 6.7	25.8 ± 7.0	26.0 ± 7.3	25.9 ± 7.5

PROMIS = Patient-Reported Outcomes Measurement Information System (0-100), with higher numbers indicating higher levels of physical functioning

PSEQ = 10-item Pain Self-Efficacy Questionnaire (0 to 60), with higher numbers indicating higher pain self-efficacy

PHQ-9 = 9-item Patient Health Questionnaire (0 to 27), with higher numbers indicating higher depressive symptoms

TSK-13 = 13-item Tampa Scale for Kinesiophobia (13 to 52), with higher numbers indicating higher fear of movement

^a^Missing data for steps per day is *n*=26 at 6 weeks, *n*=37 at 6 months, *n*=38 at 12 months, and *n*=138 at 24 months

^b^Missing data for PROMIS physical function is *n*=10 at preoperative visit, *n*=1 at 6 weeks, *n*=12 at 6 months, *n*=17 at 12 months, and *n*=123 at 24 months

^c^Missing data for PESQ is *n*=13 at preoperative visit, *n*=1 at 6 weeks, *n*=13 at 6 months, *n*=19 at 12 months, and *n*=123 at 24 months

^d^Missing data for PHQ-9 is *n*=14 at preoperative visit, *n*=1 at 6 weeks, *n*=11 at 6 months, *n*=17 at 12 months, and *n*=125 at 24 months

^e^Missing data for TSK-13 is *n*=13 at preoperative visit, *n*=1 at 6 weeks, *n*=13 at 6 months, *n*=19 at 12 months, and *n*=123 at 24 months

### Relation between physical activity and physical function

Across all the imputed datasets (M1 to M5), the range of CFI, TLI, SRMR and RMSEA values of the SEM model examining the longitudinal relationship between physical activity and physical function were 0.92 to 0.95, 0.89 to 0.94, 0.05 to 0.06, and 0.057 to 0.073, respectively, indicating the model fit was within an acceptable range (Table 3). The stability index was <0.001 with all eigenvalues within the unit circle for all five imputed datasets. The overall (pooled) SRW for the effect of physical activity on physical function ranged from 0.08 to 0.19, while the overall effect of physical function on activity ranged from non-existent to 0.14. Specifically, the effect of physical activity at 6 weeks on physical function at 6 months was 0.19 (*p*<0.001) and at 12 months physical activity on physical function at 24 months was 0.18 (*p*=0.01). The effect of physical activity at 6 months on physical function at 12 months was not statistically significant. The effect of physical function on physical activity was non-existent for the 6 weeks to 6 months and 12 months to 24 months models. However, the SRW for effect of physical function at 6 months on physical activity at 12 months was 0.14 (*p*-value <0.01). The effects of physical activity on physical function and function on activity remained consistent after accounting for pain self-efficacy, depression, and fear of movement in the mediation models (Supplementary Tables 1-3).

**Table 3:** Standardized regression weights (SRW) and standard errors (SE) for SEM models of physical activity and physical function from 6 weeks to 24 months after lumbar spine surgery (*N*=248)

	SRW (SE)					Overall	
Pathway	M1	M2	M3	M4	M5	SRW (95% CI)	*p*-value
***6 weeks to 6 months***							
PA → PA	1.21(0.11)	1.06(0.09)	1.10(0.09)	1.07(0.09)	1.14(0.10)	1.12(0.93,1.31)	0.0001
PF → PA	n.s.	n.s.	n.s.	n.s.	n.s.	n.s.	n.s.
PF → PF	1.33(0.20)	1.14(0.17)	1.21(0.16)	1.08(0.16)	1.14(0.16)	1.18(0.85,1.52)	0.0001
PA → PF	0.20(0.05)	0.18(0.05)	0.16(0.05)	0.23(0.05)	0.20(0.05)	0.19(0.09,0.30)	0.0006
***6 months to 12 months***							
PA →PA	0.77(0.08)	0.70(0.08)	0.80(0.08)	0.75(0.09)	0.73(0.09)	0.75(0.59,0.91)	0.0001
PF → PA	0.13(0.04)	0.10(0.05)	0.14(0.04)	0.17(0.04)	0.15(0.05)	0.14(0.05,0.22)	0.0062
PF → PF	0.85(0.09)	0.93(0.09)	0.86(0.09)	0.94(0.11)	0.87(0.10)	0.89(0.70,1.07)	0.0001
PA → PF	0.08(0.06)	0.07(0.05)	0.09(0.06)	0.05(0.06)	0.10(0.06)	0.08(-0.03,0.19)	0.1728
***12 months to 24 months***							
PA → PA	0.99(0.06)	0.82(0.07)	0.98(0.06)	0.95(0.06)	0.77(0.07)	0.90(0.78,1.02)	0.0001
PF → PA	n.s.	n.s.	n.s.	n.s.	n.s.	n.s.	n.s.
PF → PF	0.77(0.07)	0.85(0.07)	0.68(0.08)	0.74(0.07)	0.74(0.07)	0.76(0.62,0.90)	0.0001
PA → PF	0.20(0.06)	0.12(0.06)	0.18(0.06)	0.20(0.06)	0.21(0.05)	0.18(0.07,0.30)	0.0102
***Fit statistics***							
CFI	0.94	0.92	0.94	0.93	0.95		
TLI	0.92	0.89	0.92	0.91	0.94		
RMSEA	0.065	0.073	0.067	0.070	0.057		
SRMR	0.06	0.06	0.05	0.05	0.05		
Stability index	<0.001	<0.001	<0.001	<0.001	<0.001		

M1-M5 indicate the models for the five multiple imputation datasets

*PA* physical activity as measured by accelerometer (average steps per day); *PF* Physical function as measured by PROMIS scores; *CFI* Comparative Fit Index; *TLI* Tucker-Lewis Index; *SRMR* standardized root mean square residual; *RMSEA* root mean square error of approximation; *n.s.* not statistically significant

### Psychosocial factors as mediators

Across all the imputed datasets (M1 to M5), the range of the CFI, TLI, SRMR and RMSEA for the three SEM models examining the longitudinal relationship between physical activity and physical function and each of the psychosocial variables were 0.81 to 0.88, 0.76 to 0.86, 0.08 to 0.10 and 0.080 to 0.106, respectively, indicating that fit values were not within an acceptable range (Supplemental Table 1-3). However, the stability index of these three SEM models across all the imputed datasets was <0.001, indicating that the models were stable. Depression and fear of movement at 6 months mediated 3.4% and 5.4% of the effect of physical activity at 6 weeks on physical function at 12 months, respectively (Table 4). Pain self-efficacy was not a statistically significant mediator. E-values, measure of sensitivity to potentially unobserved confounders for mediation co-efficients ranged between 1.17 and 1.25. There is high likelihood that unobserved covariates would alter these results, which is expected given the small mediation effects and observational nature of study.

**Table 4:** Standardized regression weights (SRW) to quantify the role of psychosocial factors as mediators of the relation between physical activity and physical function following spine surgery

	SRW					Overall		
Mediators	M1	M2	M3	M4	M5	SRW	*p*-value	Mediation effect
**Pain Self-Efficacy (PSE)**								
T1PA → T3 PF	0.28	0.27	0.28	0.30	0.31	0.29	0.0001	0%
T1 PA → T2 PSE	n.s.	n.s.	n.s.	n.s.	n.s.	n.s.	n.s.	
T2 PSE → T3 PF	0.15	0.20	0.12	0.17	0.12	0.15	0.017	
T2 PA → T4 PF	0.24	0.19	0.23	0.25	0.29	0.24	0.0001	0%
T2 PA → T3 PSE	n.s.	n.s.	n.s.	n.s.	n.s.	n.s.	n.s.	
T3 PSE → T4 PF	0.11	0.12	0.14	0.22	0.03	0.13	0.13	
**Depression (Dep)**								
T1 PA → T3 PF	0.28	0.26	0.30	0.29	0.29	0.28	0.0001	3.43%
T1 PA → T2 Dep	-0.09	-0.08	-0.09	-0.07	-0.09	-0.08	0.108	
T2 Dep → T3 PF	-0.12	-0.19	-0.12	-0.10	-0.09	-0.12	0.0266	
T2 PA → T4 PF	0.24	0.18	0.24	0.21	0.24	0.22	0.0001	0%
T2 PA → T3 Dep	n.s.	n.s.	n.s.	n.s.	n.s.	n.s.	n.s.	
T3 Dep → T4 PF	n.s.	n.s.	n.s.	n.s.	n.s.	n.s.	n.s.	
**Fear of Movement (Fear)**								
T1 PA → T3 PF	0.28	0.27	0.28	0.30	0.32	0.29	0.0001	5.38%
T1 PA → T2 Fear	-0.12	-0.14	-0.11	-0.13	-0.13	-0.12	0.0092	
T2 Fear → T3 PF	-0.13	-0.12	-0.11	-0.15	-0.13	-0.13	0.0104	
T2 PA → T4 PF	0.23	0.18	0.23	0.22	0.27	0.22	0.0001	0%
T2 PA → T3 Fear	n.s.	n.s.	n.s.	n.s.	n.s.	n.s.	n.s.	
T3 Fear → T4 PF	n.s.	n.s.	n.s.	n.s.	n.s.	n.s.	n.s.	

T1= 6 weeks, T2 = 6 months, T3 = 12 months, and T4 = 24 months following spine surgery

M1-M5 indicate the models for the five multiple imputation datasets

*PA* physical activity as measured by accelerometer (average steps per day), *PF* Physical function as measured by PROMIS scores, *PSE* Pain self-efficacy as measured by Pain Self-Efficacy Questionnaire, *Dep* Depressive symptoms measured using Patient Health Questionnaire-9, *Fear* Fear of movement as measured by 13-item Tampa Scale for Kinesiophobia, *CFI* Comparative Fit Index, *TLI* Tucker-Lewis Index, *SRMR* standardized root mean square residual, *RMSEA* root mean square error of approximation, *n.s.* not statistically significant

## Discussion

This study used SEM statistical techniques to determine the longitudinal postoperative relationship between physical activity, psychosocial factors, and physical function in patients undergoing lumbar spine surgery. Models demonstrated that the relation of postoperative physical activity (i.e., steps per day) to physical function over time was stronger compared to the physical function to physical activity relationship. However, findings do not support a strong contribution of pain self-efficacy, depression, and fear of movement to this postoperative relationship.

Specifically, stable SEM models suggested that depression and fear of movement at 6 months mediated, to a minimal degree, the effect of physical activity at 6 weeks on physical function at 12 months. Pain self-efficacy was not found to be an important mediator of the physical activity-physical function relationship. Future research is needed to examine whether promoting physical activity during the early postoperative period leads to improvement in long-term physical function.

Our findings suggest that the relation of physical activity to function was stronger over time compared to the function to activity relationship. This finding is consistent with prior studies conducted in adults with musculoskeletal pain [8, 10, 62, 63]. Engaging in physical activity that is feasible and accessible to patients, such as walking, may preserve or improve lower-extremity muscle strength [64], reduce symptoms, and positively impact opioid use [8, 9]. Walking, in particular, provides physical and mental health benefits [63] and has been found to be associated with a lower risk of functional limitation [10], hospital re-admission [65], length of hospital stay [66] and mortality [67] in adults with musculoskeletal pain who are managed operatively or non-operatively. These positive effects in turn may explain why taking more steps per day has a beneficial effect on the broader construct of physical function following lumbar spine surgery. Intervention trials after joint arthroplasty suggest the use of wearable technology and steps per day goals may be feasible and an effective strategy to improve physical activity after orthopaedic surgery [68–70]. Further research is needed to investigate whether a structured walking program early after surgery has the potential to improve physical activity and long-term physical function in patients undergoing lumbar spine surgery.

The hypothesis that psychosocial factors of pain self-efficacy, depression and fear of movement would impact the physical activity-physical function relationship was partially supported. Results suggest that depression and fear of movement have a mediation effect between 6 weeks and 12 months after surgery. However, the effects found in our study were small, i.e., mediation effect were 3.4% and 5.4%. Prior studies in older adults have suggested that engaging in physical activity promotes psychosocial well-being and lowers the risk of developing depressive symptoms [63, 71] and influences self-efficacy [72]. Our negative findings may be due to the low psychosocial risk and limited variability in postoperative PSEQ, PHQ-9, and TSK-13 scores found within our study population. Future studies are needed to investigate the meditation effect of these psychosocial factors in patients who are identified as high-risk using validated psychosocial screening instruments.

In this study, mediators of depression and fear of movement were based on the well-established Fear Avoidance Model [73, 74]. Prior literature provides support for these psychosocial constructs as independent predictors of poor outcomes following spine surgery.^18-22^ However, pain catatrophizing, an important component of the Fear Avoidance Model, was not assessed in the current study. Coronado et al [75] and others [76–78] have found that pain catastrophizing is an important predictor of long-term patient-reported health outcomes of pain and disability after lumbar spine surgery [75–78]. An important consideration is that the Fear Avoidance Model may not fully explain the physical activity-physical function relationship. Some studies have shown that fear-related processes may not be applicable in adults with chronic pain [79, 80] and have postulated that broader models are needed that include positive psychosocial factors [80]. One such factor may be resilience, which has the potential to positively impact long-term physical function after spine surgery [22]. While our study assessed the positive psychosocial construct of self-efficacy, the instrument chosen was focused on a patient’s ability to perform an activity despite pain (i.e., pain self-efficacy) [39]. Other self-efficacy measures related to exercise or walking may be more appropriate for examining the mediation effect of physical activity on function in older adults [26, 27]. Therefore, additional work is needed to identify the psychosocial factors, both negative and positive, that have a role in how physical activity impacts function in patients after lumbar spine surgery.

The findings of this study should be viewed in the light of the following limitation. The variability in the chosen psychosocial factors was minimal, and the overall analytical sample represented a low to medium risk psychosocial profile. Evidence suggests that patients at high-risk for poor outcomes should be targeted for a biopsychosocial management approach [81, 82]; our findings may not be generalizable for those at highest psychosocial risk. Approximately, 50% of the physical activity and patient-reported outcomes were missing at the 24-month time-point. To address this limitation, we used multiple imputation techniques that leveraged a longitudinal study design, and less than 15% of physical activity and patient-reported outcomes data were missing at prior timepoints [83]. Further, we conducted sensitivity analyses with complete data to confirm findings, and used separate models for the 12 and 24-month outcomes. The statistical literature suggests that when greater than 40% of data are missing for important variables, the results of the study should be considered as hypothesis generating [84, 85]. Therefore, the findings of this study should be viewed as a first step toward testing a conceptual model of postoperative recovery after lumbar spine surgery.

Data for this study were obtained from a prior randomized controlled trial study. While group and other potential covariates, which were identified from literature and our preliminary work (separate bivariate analysis) were accounted for in all analyses, there is the potential for residual confounding. Given the observational nature of the study, we acknowledge that causality with certainty cannot be concluded. Therefore, in this study, we have used the term “relationship” as opposed to “causation” since the experimental data needed to test the causality is difficult to collect. The fit indexes for the mediation models were not within acceptable range; however, the stability index indicated that all models were stable. Finally, we acknowledge that a sample size greater than 250 is preferred to identify good fit indices for SEM models [55]. Future research with a larger sample size is needed to validate this conceptual model of postoperative recovery after spine surgery.

## Conclusion

The relation of postoperative physical activity to function over time was stronger compared to the function to activity relationship. Early physical activity after surgery was related to long-term physical function in patients undergoing lumbar spine surgery. Depression and fear of movement demonstrated a very small mediating effect (3.4% and 5.4%) during the first 12 months. However, given the observational nature of this study, causation must not be inferred and these study findings should be viewed as a first step toward testing a conceptual model of postoperative recovery after lumbar spine surgery. Future work should consider investigating other potential mediating factors, such as pain catatrophizing, and broader psychosocial models that include positive psychosocial constructs of resilience and exercise/walking self-efficacy.

## Supplementary Information

**Additional file 1: Supplementary Table 1.** Standardized regression weights (SRW) and standard errors (SE) for SEM mediation models of physical activity, physical function, and pain self-efficacy from 6 weeks to 24 months after lumbar spine surgery (N=248). **Supplementary Table 2.** Standardized regression weights (SRW) and standard errors (SE) for SEM mediation models of physical activity, physical function, and depression from 6 weeks to 24 months after lumbar spine surgery (N=248). **Supplementary Table 3.** Standardized regression weights (SRW) and standard errors (SE) for SEM mediation models of physical activity, physical function, and fear of movement from 6 weeks to 24 months after lumbar spine surgery (N=248).

## Data Availability

The datasets used and/or analyzed during the current study are available by reasonable request and at the discretion of the corresponding author.

## Abbreviations

CBPT: Cognitive-behavioral based physical therapy
CAT: the computerized adaptive test
PROMIS: Patient-Reported Outcomes Measurement Information System
PSEQ: Pain Self-Efficacy Questionnaire
PHQ-9: Patient Health Questionnaire-9
TSK-13: 13-item Tampa Scale for Kinesiophobia
CFI: Comparative Fit Index
TLI: Tucker-Lewis Index (TLI)
SRMR: Standardized root mean squared residual
RMSEA: Root Mean Square Error of Approximation
SEM: Structural equation modeling
M1 to M5: Five imputed datasets
