# Supplementary material for: Role of psychosocial factors on the effect of physical activity on physical function in patients after lumbar spine surgery
Source: BMC Musculoskelet Disord. 2021 Oct 18;22:883. doi: 10.1186/s12891-021-04622-w (PMC8522146; doi:10.1186/s12891-021-04622-w)
Supplement: Supplementary file 1 — Additional file 1: Supplementary Table 1. Standardized regression weights (SRW) and standard errors (SE) for SEM mediation models of physical activity, physical function, and pain self-efficacy from 6 weeks to 24 months after lumbar spine surgery (N=248). Supplementary Table 2. Standardized regression weights (SRW) and standard errors (SE) for SEM mediation models of physical activity, physical function, and depression from 6 weeks to 24 months after lumbar spine surgery (N=248). Supplementary Table 3. Standardized regression weights (SRW) and standard errors (SE) for SEM mediation models of physical activity, physical function, and fear of movement from 6 weeks to 24 months after lumbar spine surgery (N=248). [file 12891_2021_4622_MOESM1_ESM.docx]

**Supplementary Table 1.** Standardized regression weights (SRW) and standard errors (SE) for SEM mediation models of physical activity, physical function, and pain self-efficacy from 6 weeks to 24 months after lumbar spine surgery (N=248).

|  | **SRW (SE)** | | | | | **Overall** | |
| --- | --- | --- | --- | --- | --- | --- | --- |
| **Pathway** | **M1** | **M2** | **M3** | **M4** | **M5** | **SRW** | **p-value** |
| ***6 weeks to 6 months*** | | | | | | | |
| PA → PA | 1.11(0.04) | 1.06(0.09) | 1.10(0.09) | 1.07(0.09) | 1.14(0.10) | 0.10 | 0.0001 |
| PF → PA | n.s. | n.s. | n.s. | n.s. | n.s. | n.s. | n.s. |
| PSE → PA | n.s. | n.s. | n.s. | n.s. | n.s. | n.s. | n.s. |
| PF → PF | 1.13(0.07) | 1.06(0.16) | 1.16(0.17) | 1.07(0.17) | 1.11(0.17) | 1.10 | 0.0001 |
| PA → PF | 0.18(0.02) | 0.17(0.06) | 0.16(0.06) | 0.22(0.06) | 0.18(0.06) | 0.18 | 0.0016 |
| PSE → PF | 0.12(0.03) | 0.13(0.06) | 0.13(0.06) | 0.08(0.06) | 0.13(0.06) | 0.12 | 0.0438 |
| PSE → PSE | 0.92(0.05) | 1.01(0.13) | 0.80(0.10) | 0.95(0.12) | 0.95(0.12) | 0.93 | 0.0001 |
| PA → PSE | n.s. | n.s. | n.s. | n.s. | n.s. | n.s. | n.s. |
| PF → PSE | 0.18(0.03) | 0.20(0.06) | 0.14(0.06) | 0.16(0.06) | 0.18(0.06) | 0.17 | 0.0074 |
| ***6 months to 12 months*** | | | | | | | |
| PA → PA | 0.75(0.04) | 0.70(0.08) | 0.80(0.08) | 0.75(0.09) | 0.72(0.09) | 0.74 | 0.0001 |
| PF → PA | 0.14(0.02) | 0.10(0.05) | 0.14(0.04) | 0.17(0.04) | 0.16(0.05) | 0.14 | 0.0062 |
| PSE → PA | n.s. | n.s. | n.s. | n.s. | n.s. | n.s. | n.s. |
| PF → PF | 0.69(0.04) | 0.68(0.09) | 0.73(0.08) | 0.68(0.09) | 0.67(0.09) | 0.69 | 0.0001 |
| PA → PF | 0.14(0.03) | 0.14(0.06) | 0.14(0.06) | 0.14(0.06) | 0.16(0.06) | 0.15 | 0.01 |
| PSE → PF | 0.15(0.02) | 0.20(0.05) | 0.12(0.06) | 0.17(0.06) | 0.12(0.06) | 0.15 | 0.017 |
| PSE → PSE | 0.82(0.04) | 0.77(0.09) | 0.82(0.12) | 0.80(0.10) | 0.86(0.10) | 0.81 | 0.0001 |
| PA → PSE | n.s. | n.s. | n.s. | n.s. | n.s. | n.s. | n.s. |
| PF → PSE | 0.11(0.02) | 0.07(0.06) | 0.17(0.05) | 0.11(0.06) | 0.11(0.06) | 0.11 | 0.0796 |
| ***12 months to 24 months*** | | | | | | | |
| PA → PA | 0.90(0.03) | 0.82(0.07) | 0.99(0.06) | 0.93(0.07) | 0.79(0.07) | 0.89 | 0.0001 |
| PF → PA | n.s. | n.s. | n.s. | n.s. | n.s. | n.s. | n.s. |
| PSE → PA | n.s. | n.s. | n.s. | n.s. | n.s. | n.s. | n.s. |
| PF → PF | 0.61(0.04) | 0.72(0.10) | 0.50(0.11) | 0.54(0.10) | 0.64(0.10) | 0.60 | 0.0001 |
| PA → PF | 0.20(0.03) | 0.13(0.06) | 0.20(0.07) | 0.23(0.06) | 0.24(0.06) | 0.20 | 0.0078 |
| PSE → PF | 0.11(0.03) | 0.12(0.06) | 0.14(0.06) | 0.22(0.06) | 0.03(0.06) | 0.13 | 0.1282 |
| PSE → PSE | 0.76(0.05) | 0.68(0.12) | 0.82(0.14) | 0.74(0.13) | 0.65(0.12) | 0.73 | 0.0001 |
| PA → PSE | n.s. | n.s. | n.s. | n.s. | n.s. | n.s. | n.s. |
| PF → PSE | 0.13(0.03) | 0.16(0.06) | 0.14(0.07) | 0.20(0.07) | 0.16(0.07) | 0.15 | 0.0214 |
| ***Fit statistics*** |  |  |  |  |  |  |  |
| CFI | 0.82 | 0.81 | 0.83 | 0.82 | 0.84 |  |  |
| TLI | 0.78 | 0.76 | 0.79 | 0.78 | 0.80 |  |  |
| RMSEA | 0.105 | 0.106 | 0.101 | 0.104 | 0.098 |  |  |
| SRMR | 0.09 | 0.09 | 0.09 | 0.09 | 0.08 |  |  |
| Stability index | <0.001 | <0.001 | <0.001 | <0.001 | <0.001 |  |  |

PA = physical activity as measured by accelerometer (average steps per day); PF=Physical function as measured by PROMIS scores; PSE=Pain self-efficacy as measured by Pain Self-Efficacy Questionnaire; CFI= Comparative Fit Index; TLI= Tucker-Lewis Index; SRMR= standardized root mean square residual; RMSEA= root mean square error of approximation; n.s.= not statistically significant. M1-M5 indicate the models for the five multiple imputation datasets

**Supplementary Table 2.** Standardized regression weights (SRW) and standard errors (SE) for SEM mediation models of physical activity, physical function, and depression from 6 weeks to 24 months after lumbar spine surgery (N=248).

|  | **SRW (SE)** | | | | | **Overall** | |
| --- | --- | --- | --- | --- | --- | --- | --- |
| **Pathway** | **M1** | **M2** | **M3** | **M4** | **M5** | **SRW** | **p-value** |
| ***6 weeks to 6 months*** | | | | | | | |
| PA → PA | 1.09(0.04) | 1.00(0.09) | 1.10(0.08) | 1.08(0.09) | 1.14(0.09) | 1.08 | 0.0001 |
| PF → PA | n.s. | n.s. | n.s. | n.s. | n.s. | n.s. | n.s. |
| DEP → PA | 0.06(0.02) | 0.05(0.04) | 0.08(0.04) | 0.07(0.04) | 0.06(0.04) | 0.06 | 0.0948 |
| PF → PF | 0.85(0.05) | 0.85(0.10) | 0.82(0.10) | 0.93(0.11) | 0.86(0.11) | 0.86 | 0.0001 |
| PA → PF | 0.11(0.03) | 0.10(0.05) | 0.13(0.06) | 0.08(0.06) | 0.10(0.06) | 0.10 | 0.0836 |
| DEP → PF | -0.12(0.02) | -0.19(0.05) | -0.12(0.05) | -0.10(0.05) | -0.09(0.05) | -0.12 | 0.0266 |
| DEP → DEP | 0.94(0.04) | 0.97(0.10) | 0.93(0.11) | 0.91(0.10) | 0.91(0.09) | 0.93 | 0.0001 |
| PA → DEP | -0.09(0.05) | -0.08(0.05) | -0.09(0.06) | -0.07(0.05) | -0.09(0.05) | -0.08 | 0.108 |
| PF → DEP | -0.11(0.03) | -0.07(0.06) | -0.10(0.06) | -0.11(0.06) | -0.11(0.06) | -0.10 | 0.0958 |
| ***6 months to 12 months*** | | | | | | | |
| PA → PA | 0.82(0.02) | 0.86(0.05) | 0.81(0.04) | 0.77(0.05) | 0.79(0.05) | 0.81 | 0.0001 |
| PF → PA | 0.13(0.02) | 0.10(0.05) | 0.14(0.04) | 0.17(0.04) | 0.15(0.04) | 0.14 | 0.009 |
| DEP → PA | n.s. | n.s. | n.s. | n.s. | n.s. | n.s. | n.s. |
| PF → PF | 0.85(0.05) | 0.85(0.10) | 0.82(0.10) | 0.93(0.11) | 0.86(0.11) | 0.86 | 0.0001 |
| PA → PF | 0.11(0.03) | 0.10(0.05) | 0.13(0.06) | 0.08(0.06) | 0.10(0.06) | 0.10 | 0.0836 |
| DEP → PF | -0.12(0.02) | -0.19(0.05) | -0.12(0.05) | -0.10(0.05) | -0.09(0.05) | -0.12 | 0.0266 |
| DEP → DEP | 0.81(0.04) | 0.80(0.08) | 0.80(0.09) | 0.85(0.09) | 0.82(0.09) | 0.82 | 0.0001 |
| PA → DEP | n.s. | n.s. | n.s. | n.s. | n.s. | n.s. | n.s. |
| PF → DEP | -0.16(0.02) | -0.14(0.05) | -0.19(0.05) | -0.14(0.05) | -0.16(0.05) | -0.16 | 0.0018 |
| ***12 months to 24 months*** | | | | | | | |
| PA → PA | 0.91(0.03) | 0.83(0.07) | 0.99(0.06) | 0.96(0.06) | 0.79(0.07) | 0.90 | 0.0001 |
| PF → PA | n.s. | n.s. | n.s. | n.s. | n.s. | n.s. | n.s. |
| DEP → PA | n.s. | n.s. | n.s. | n.s. | n.s. | n.s. | n.s. |
| PF → PF | 0.66(0.05) | 0.81(0.10) | 0.49(0.14) | 0.68(0.11) | 0.76(0.10) | 0.68 | 0.0001 |
| PA → PF | 0.20(0.03) | 0.12(0.06) | 0.22(0.07) | 0.21(0.06) | 0.21(0.06) | 0.19 | 0.0072 |
| DEP → PF | n.s. | n.s. | n.s. | n.s. | n.s. | n.s. | n.s. |
| DEP → DEP | 0.69(0.05) | 0.61(0.11) | 0.63(0.11) | 0.71(0.11) | 0.87(0.11) | 0.70 | 0.0001 |
| PA → DEP | -0.12(0.03) | -0.14(0.06) | -0.11(0.06) | -0.12(0.06) | -0.08(0.06) | -0.11 | 0.066 |
| PF → DEP | -0.13(0.03) | -0.16(0.07) | -0.13(0.07) | -0.16(0.07) | -0.08(0.07) | -0.13 | 0.072 |
| ***Fit statistics*** |  |  |  |  |  |  |  |
| CFI | 0.84 | 0.86 | 0.83 | 0.86 | 0.88 |  |  |
| TLI | 0.81 | 0.83 | 0.79 | 0.84 | 0.86 |  |  |
| SRMR | 0.07 | 0.08 | 0.08 | 0.07 | 0.07 |  |  |
| RMSEA | 0.098 | 0.087 | 0.102 | 0.088 | 0.080 |  |  |
| Stability index | <0.001 | <0.001 | <0.001 | <0.001 | <0.001 |  |  |

PA = physical activity as measured by accelerometer (average steps per day); PF=Physical function as measured by PROMIS scores; DEP = Depressive symptoms measured using Patient Health Questionnaire-9; CFI= Comparative Fit Index; TLI= Tucker-Lewis Index; SRMR= standardized root mean square residual; RMSEA= root mean square error of approximation; n.s.= not statistically significant. M1-M5 indicate the models for the five multiple imputation datasets.

**Supplementary Table 3.** Standardized regression weights (SRW) and standard errors (SE) for SEM mediation models of physical activity, physical function, and fear of movement from 6 weeks to 24 months after lumbar spine surgery (N=248).

|  | **SRW (SE)** | | | | | **Overall** | |
| --- | --- | --- | --- | --- | --- | --- | --- |
| **Pathway** | **M1** | **M2** | **M3** | **M4** | **M5** | **SRW** | **p-value** |
| ***6 weeks to 6 months*** | | | | | | | |
| PA → PA | 1.11(0.04) | 1.07(0.09) | 1.11(0.09) | 1.09(0.09) | 1.15(0.10) | 1.11 | 0.0001 |
| PF → PA | n.s. | n.s. | n.s. | n.s. | n.s. | n.s. | n.s. |
| Fear → PA | n.s. | n.s. | n.s. | n.s. | n.s. | n.s. | n.s. |
| PF → PF | 1.12(0.07) | 1.07(0.15) | 1.15(0.15) | 1.03(0.15) | 1.12(0.15) | 1.10 | 0.0001 |
| PA → PF | 0.19(0.02) | 0.18(0.06) | 0.17(0.06) | 0.23(0.06) | 0.19(0.06) | 0.19 | 0.0008 |
| Fear → PF | n.s. | n.s. | n.s. | n.s. | n.s. | n.s. | n.s. |
| Fear → Fear | 0.90(0.04) | 0.91(0.08) | 0.97(0.08) | 0.89(0.08) | 0.94(0.08) | 0.93 | 0.0001 |
| PA → Fear | -0.12(0.02) | -0.14(0.05) | -0.11(0.05) | -0.13(0.05) | -0.13(0.05) | -0.12 | 0.0092 |
| PF → Fear | n.s. | n.s. | n.s. | n.s. | n.s. | n.s. | n.s. |
| ***6 months to 12 months*** | | | | | | | |
| PA → PA | 0.74(0.03) | 0.70(0.08) | 0.79(0.08) | 0.73(0.09) | 0.73(0.09) | 0.74 | 0.0001 |
| PF → PA | 0.14(0.02) | 0.10(0.05) | 0.14(0.04) | 0.18(0.04) | 0.15(0.05) | 0.14 | 0.0056 |
| Fear → PA | n.s. | n.s. | n.s. | n.s. | n.s. | n.s. | n.s. |
| PF → PF | 0.76(0.03) | 0.78(0.08) | 0.78(0.07) | 0.75(0.08) | 0.70(0.08) | 0.75 | 0.0001 |
| PA → PF | 0.11(0.02) | 0.10(0.05) | 0.12(0.06) | 0.10(0.06) | 0.15(0.06) | 0.12 | 0.0416 |
| Fear → PF | -0.13(0.02) | -0.12(0.05) | -0.11(0.05) | -0.15(0.05) | -0.13(0.05) | -0.13 | 0.0104 |
| Fear → Fear | 0.89(0.03) | 0.95(0.08) | 0.85(0.07) | 0.90(0.08) | 0.90(0.08) | 0.90 | 0.0001 |
| PA → Fear | n.s. | n.s. | n.s. | n.s. | n.s. | n.s. | n.s. |
| PF → Fear | -0.13(0.02) | -0.09(0.05) | -0.15(0.04) | -0.11(0.05) | -0.11(0.05) | -0.12 | 0.02 |
| ***12 months to 24 months*** | | | | | | | |
| PA → PA | 0.90(0.03) | 0.81(0.07) | 0.98(0.06) | 0.95(0.06) | 0.77(0.07) | 0.88 | 0.0001 |
| PF → PA | n.s. | n.s. | n.s. | n.s. | n.s. | n.s. | n.s. |
| Fear → PA | n.s. | n.s. | n.s. | n.s. | n.s. | n.s. | n.s. |
| PF → PF | 0.72(0.03) | 0.82(0.07) | 0.65(0.08) | 0.72(0.07) | 0.71(0.07) | 0.72 | 0.0001 |
| PA → PF | 0.20(0.03) | 0.13(0.06) | 0.19(0.06) | 0.21(0.06) | 0.23(0.05) | 0.19 | 0.0056 |
| Fear → PF | n.s. | n.s. | n.s. | n.s. | n.s. | n.s. | n.s. |
| Fear → Fear | 0.77(0.02) | 0.78(0.05) | 0.75(0.05) | 0.80(0.05) | 0.080(0.05) | 0.78 | 0.0001 |
| PA → Fear | n.s. | n.s. | n.s. | n.s. | n.s. | n.s. | n.s. |
| PF → Fear | n.s. | n.s. | n.s. | n.s. | n.s. | n.s. | n.s. |
| ***Fit statistics*** |  |  |  |  |  |  |  |
| CFI | 0.85 | 0.87 | 0.88 | 0.88 | 0.86 |  |  |
| TLI | 0.82 | 0.84 | 0.85 | 0.86 | 0.83 |  |  |
| SRMR | 0.08 | 0.08 | 0.07 | 0.08 | 0.08 |  |  |
| RMSEA | 0.096 | 0.088 | 0.087 | 0.085 | 0.093 |  |  |
| Stability index | <0.001 | <0.001 | <0.001 | <0.001 | <0.001 |  |  |

PA = physical activity as measured by accelerometer (average steps per day); PF=Physical function as measured by PROMIS scores; Fear=Fear of movement as measured by 13-item Tampa Scale for Kinesiophobia; CFI= Comparative Fit Index; TLI= Tucker-Lewis Index; SRMR= standardized root mean square residual; RMSEA= root mean square error of approximation; n.s.= not statistically significant. M1-M5 indicate the models for the five multiple imputation datasets.
